# Evaluating Fiscal Credibility Through the Lens of Durability: Value Decay in AI‐Enabled Clinical Documentation

**DOI:** 10.1111/jep.70715

**Published:** 2026-11-03

**Authors:** Samuel Atiku, Olufisayo Olakotan

**Affiliations:** ^1^ Research Services Aston University Birmingham UK; ^2^ Digital Technology and Innovation University of Staffordshire UK; ^3^ Department of Neonatology, Women and Children's Directorate University Hospitals Leicester NHS Trust Leicester UK

**Keywords:** artificial intelligence, clinical documentation, durability, fiscal credibility, netnography, value decay, workforce burnout

## Abstract

**Rationale, Aims, and Objectives:**

AI‐enabled clinical documentation tools are deployed to mitigate workforce burnout, yet technical efficiency observed in short‐term evaluations frequently fails to translate into sustained institutional value. This study evaluates the fiscal credibility of these tools through the lens of durability, investigating how their value is sustained, eroded, or transformed once routine implementation is established.

**Methodology:**

We employed a netnographic design to analyse professional discourse across seven healthcare subreddits. Using a custom harvesting protocol over a 24‐month window (2024–2025), we screened 2645 text segments. A “Double‐Lock” filtration system isolated 39 high‐fidelity discursive incidents focusing on post‐implementation sustainment. Data were analysed using Reflexive Thematic Analysis informed by an abductive logic.

**Results:**

Analysis reveals that durability failure is driven by three socio‐organisational mechanisms of value decay rather than technical inadequacy. First, *Incentive Conversion* occurs when administrative actors capture efficiency gains to increase throughput via a Jevons Paradox effect, transforming workforce relief into labour intensification. Second, *Maintenance Entropy* creates a hidden maintenance debt through the gradual accumulation of unpriced articulation work. Third, *Legitimacy Collapse* occurs when the cognitive load of verification exceeds the clinician's bandwidth, leading to the rejection of the tool's epistemic authority.

**Conclusion:**

AI documentation tools risk becoming “zombie technologies”, operationally active but fiscally hollow, because institutional extraction mechanisms cannibalise their intended value. Achieving true durability requires contractually ring‐fencing efficiency dividends for workforce recovery and explicitly pricing the shadow labour of maintenance and human‐in‐the‐loop verification. Because fiscal and ethical judgements may conflict, we recommend that their oversight operate as separate and independent functions. These findings have direct implications for health systems reform, particularly procurement governance, productivity policy, and workforce sustainability strategies for digital health.

## 1 Introduction

Digital health technologies, and particularly artificial intelligence‐enabled clinical documentation tools, are increasingly promoted as a solution to the crisis of healthcare workforce sustainability. Rising administrative burden, intensifying documentation requirements, and widespread clinician burnout have created a policy and managerial climate in which automation is framed as both inevitable and urgent [1, 2]. Ambient AI scribes and automated documentation platforms are positioned not simply as efficiency aids, but as instruments of workforce rescue that promise to return time to clinicians, restore the therapeutic relationship, and stabilise fragile health systems. Early‐stage evaluations and pilot studies appear to support this narrative, reporting reductions in documentation time, improvements in perceived efficiency, and short‐term gains in user satisfaction following implementation [3].

Despite this optimism, a persistent paradox characterises the trajectory of digital health innovation. Interventions that demonstrate technical efficacy and positive reception during pilots frequently fail to persist in routine practice. The history of health information technology is marked by systems that were successfully implemented yet later abandoned, scaled back, or quietly discontinued once external funding, leadership attention, or implementation support was withdrawn [4]. This recurring pattern, often described as a pilot‐to‐scale gap, indicates that the drivers of initial adoption differ fundamentally from the mechanisms required for long‐term sustainment.

This study addresses this gap by examining the socio‐organisational dynamics of durability. Durability is defined here not as the technical stability or uptime of software, but as the capacity of an intervention to generate sustained and institutionally recognised value under conditions of routine operation. Fiscal credibility, in turn, concerns whether the value claimed for a technology at procurement can reasonably be expected to persist once it is embedded in routine care, rather than only during a supported pilot. While existing research has focused heavily on proximal determinants of adoption such as usability, training, and short‐term workflow impact [5], far less attention has been paid to the distal processes through which value decays, mutates, or is reallocated over time. In particular, there is limited empirical understanding of what happens after AI documentation tools become embedded in everyday clinical work and subject to ordinary organisational pressures.

This study asks the following question: How is the value of AI‐enabled clinical documentation sustained, eroded, or transformed once routine implementation is established?

We address this question through a netnographic analysis of professional discourse occurring in the backstage spaces of healthcare operations. Online professional communities provide access to informal sensemaking around implementation failures, maintenance burdens, governance frictions, and renewal decisions that are rarely visible in formal evaluations or vendor‐led studies [6, 7]. Focusing on these spaces allows examination of how durability is negotiated in practice rather than assumed through adoption metrics.

The analysis suggests that durability failure is rarely attributable to simple technical inadequacy. Instead, it reflects an extractive institutional logic that reallocates the gains of automation away from clinical labour. Efficiency gains promised to clinicians are frequently converted into expectations of increased throughput, while the unpriced labour of maintenance, verification, and error correction gradually erodes net utility. At the same time, the cognitive burden of supervising probabilistic systems accumulates, contributing to trust erosion and professional fatigue. Because clinicians remain responsible for records they increasingly do not author, this erosion also carries an ethical dimension, since accountability may remain formally assigned while the capacity to exercise it weakens. Without a restructuring of the socio‐technical contract governing automation, AI documentation tools risk becoming zombie technologies that remain operational yet fail to sustain institutional legitimacy or workforce benefit. The final sections consider what this implies for governance and argue that fiscal and ethical oversight of these tools should operate as separate and independent functions.

To anchor this evaluation, Table 1 defines the key theoretical constructs used to map the specific socio‐organisational mechanisms of value decay identified in this study.

**Table 1:** Definitions of key socio‐organisational mechanisms of value decay.

Construct	Definition	Operational mechanism (manifestation in data)
Durability	The capacity of a digital health intervention to generate sustained, institutionally recognised value under conditions of routine operation, distinct from technical stability or uptime.	Continued voluntary use; renewal of contracts; integration into “standard work” without requiring “hyper‐care” support.
Incentive conversion	The process by which efficiency gains (time saved by automation) are captured by administrative actors to increase production targets rather than being returned to the workforce as relief.	The Jevons Paradox: Immediate reallocation of “saved” time to additional patient slots; the “ratcheting” of baseline productivity expectations.
Maintenance entropy	The gradual accumulation of unpriced technical and workflow friction that emerges after the withdrawal of implementation support, degrading the “seamlessness” of the tool.	Articulation Work: Template fragmentation; broken EHR integrations; manual re‐assembly of “ambient” text; the need for constant “gluing” work to keep the system functional.
Legitimacy collapse	The exhaustion of professional trust that occurs when the cognitive load required to verify and correct algorithmic outputs exceeds the user's available mental bandwidth (“Verification Budget”).	The Moral Crumple Zone: Rejection of the tool's epistemic authority; regarding outputs as “garbage”; “shadow workload” of hyper‐vigilance regarding hallucinations.

## 2 Methodology

### 2.1 Research Design and Rationale

To answer the central research question, how is the value of AI‐enabled clinical documentation sustained, eroded, or transformed once routine implementation is established, we employed a netnographic research design. This approach suits the object of enquiry because durability failures such as quiet abandonment, non renewal, and the gradual accumulation of maintenance debt rarely appear in formal organisational records. These dynamics are discussed more candidly in backstage professional spaces where participants compare experiences, surface frustrations, and share workarounds [7]. The analysis therefore focused on peer‐to‐peer discourse in order to identify the informal governance practices and everyday frictions that become visible only after the initial implementation period has passed.

### 2.2 Data Collection and Sampling Strategy

We collected data from seven professional subreddits (r/healthIT, r/HealthInformatics, r/medicine, r/Residency, r/FamilyMedicine, r/emergencymedicine, r/nursing) chosen for their density of cross‐hierarchical exchange regarding health system governance. The corpus was bounded to discussions posted between January 2024 and December 2025 to reflect contemporary deployment conditions. Rather than conducting a longitudinal study of fixed participants, we treated temporality as an analytic property of the discourse. We utilised a custom computational harvesting instrument to identify threads containing substantive references to both algorithmic healthcare systems and post‐deployment experiences (e.g., “after rollout,” “did not renew,” “maintenance costs”), effectively filtering out speculative commentary to focus on the lived reality of sustainment.

### 2.3 Data Analysis

We analysed the data using Reflexive Thematic Analysis [8]informed by an abductive logic [9]. This combination helped us attend to latent socio‐organisational mechanisms expressed through professional discourse, while remaining open to unexpected patterns that extended our prior assumptions about fiscal credibility.

We conducted the analysis through three recursive phases. We began with deep familiarisation. We read the full thread context for each flagged durability incident to understand the emotional tone and organisational rhythm of the discussions. We defined the unit of analysis as the discursive incident, meaning a discrete segment of text that articulated a specific friction, decision, or adaptation related to long‐term use of the technology. We then carried out line‐by‐line coding. Our initial codes were semantic and descriptive, staying close to participants' own language, including phrases such as template fragmentation, admin increased quota, and hallucination fatigue. We grouped these labels iteratively into conceptual categories. We clustered distinct codes describing software updates, broken integrations, and version regressions under Technical Friction.

We then interrogated these categories to identify the mechanisms producing the observed outcomes, and we synthesised them into the three higher‐order themes presented in this paper. We reframed descriptive groupings as theoretical constructs through interpretation. We interpreted Technical Friction as Maintenance Entropy, understood as the structural accumulation of unpriced labour, and we interpreted volume pressures as Incentive Conversion. We strengthened rigour through negative case analysis. We actively sought incidents where durability was maintained, which helped us specify the conditions under which the identified mechanisms did not hold.

### 2.4 Ethical Considerations

Ethical approval was obtained from Aston University. We followed established ethical guidance for internet‐mediated research. We drew data exclusively from publicly accessible online communities requiring no registration. We adopted a passive observational stance, anonymised all usernames and institutional identifiers, and slightly edited quotations to ensure clarity while preserving the integrity of the professional discourse.

## 3 Results

### 3.1 Data Harvesting and Corpus Composition

The automated harvesting protocol, executed over a 24‐month sampling window (January 2024–December 2025), scanned a total of 2645 potential discursive segments across seven professional domains. To isolate genuine durability signal from general administrative noise, the “Double‐Lock” filtration system was applied. This process excluded 98.5% of the raw corpus, effectively filtering out theoretical AI speculation, vendor marketing, and unrelated clinical chatter. The progression from raw harvest through candidate screening to final inclusion is summarised in Table 2.

**Table 2:** Composition of the durability corpus.

Data stage	*N* (Count)	Description
Raw harvest	2645	Total comments/posts scanned containing AI keywords + Org context.
Filtered candidates	420	Segments matching specific query combinations (e.g., “AI scribe” + “renewal”).
Final inclusion	39	Verified incidents containing both Temporal Markers and Durability Constructs.
Source threads	5	Distinct “Mega‐Threads” where longitudinal debate occurred.

The final analytical corpus consisted of 39 high‐fidelity discursive incidents derived from 5 distinct thread clusters. These incidents represented deep, longitudinal engagement with AI documentation tools, specifically focusing on the post‐implementation phase. The distribution of these incidents was uneven across professional roles, with the highest density of durability failures reported in r/nursing (39% of incidents) and r/Residency (25%), suggesting that the friction of maintenance and throughput pressure is felt most acutely by frontline staff rather than IT leadership.

The qualitative analysis of these high‐fidelity incidents reveals that the failure of durability is rarely a consequence of simple technical inadequacy. Instead, the data suggest that value decay is driven by three emergent socio‐organisational mechanisms: the reconfiguration of efficiency into throughput pressure (Incentive Conversion), the gradual accumulation of unpriced workflow friction (Maintenance Entropy), and the exhaustion of professional trust (Legitimacy Collapse).

### 3.2 Incentive Conversion: The Cannibalisation of Workforce Relief

The primary narrative driving the adoption of AI scribes is the promise of “time banking”, returning hours to the clinician to alleviate burnout. However, our data reveal a pervasive “Jevons Paradox” effect, whereby the efficiency gains generated by the technology are immediately captured by administrative actors to increase production targets. Rather than functioning as a tool for workforce relief, the AI scribe becomes a mechanism for intensifying labour density.

Participants described a bait‐and‐switch dynamic in which the introduction of the tool served as the pretext for ratcheting up patient quotas. One clinician, describing the terms of a new role at an urban FQHC, noted that the provision of AI tools was explicitly coupled with an administrative demand to “eventually see at least 21 patients a day, 5 days a week.” Here, the technology does not reduce the burden of work. Instead, it compresses the time available to perform it, transforming the clinical encounter into a high velocity assembly line.

Crucially, this conversion of time savings into throughput appears to function as a ratchet. Once increased productivity is demonstrated, it resets the institutional baseline. Participants warned that “expectations” must be managed aggressively “right from the jump”, because once the new volume is established, “it's so much harder to walk them back afterwards.” This suggests that durability is undermined by a misalignment of incentives. The “fiscal value” of the programme is realised by the payer through increased billable volume, while the “cost” is externalised onto the clinician in the form of cognitive intensity. Under these conditions, the tool is eventually rejected or resented not because it fails to write notes, but because it succeeds in eroding the pauses that make clinical work sustainable.

### 3.3 Maintenance Entropy and the Re‐Emergence of Friction

While implementation pilots are typically characterised by “hyper care” support and optimised configurations, the longitudinal data reveal a phenomenon of Maintenance Entropy. As the novelty of the intervention fades and support teams withdraw, the “seamless” integration between the AI and the Electronic Health Record begins to degrade. The promise of “ambient” documentation dissolves into the mundane reality of copy‐and‐paste friction and manual reassembly.

Far from being a static asset, the AI model and its integration points require continuous, unpriced labour to remain functional. Users reported that the output often failed to map cleanly onto rigid institutional templates, noting that while the text appeared organised, “sections of SOAP note are broken up”, making it “actually a pain in the butt to copy all the sections I need.” This re‐emergence of manual friction, the “gluing work” required to bridge the AI and the Electronic Health Record, undermines the core value proposition of the tool.

Furthermore, this maintenance burden represents a hidden fiscal liability that is rarely accounted for in procurement models. As one respondent with experience in implementation management reflected, the critical threat to durability is the unbudgeted cost of adaptation. They noted that “how much money to maintain AI over the long‐term is an interesting question”, particularly when accounting for the continuous need to fix bugs, update models “for new research”, and handle “uncertain clinical situations” that the algorithm cannot parse. When these long tail costs are ignored, the system creates a “maintenance debt” that is eventually paid by the frontline user in the form of broken workflows and wasted time.

### 3.4 Legitimacy Collapse and the “Verification Budget”

Finally, the durability of AI documentation is threatened by the fragility of professional trust. Our analysis suggests that clinicians possess a finite “verification budget”, which is a limit to the amount of cognitive load they are willing to expend policing the machine. When the frequency of hallucinations or contextual errors breaches this threshold, Legitimacy Collapse occurs.

This collapse is often binary and emotional. Users do not merely critique the error rate. They reject the epistemic authority of the tool entirely. One participant described the output as “untrustworthy garbage”, stating that they “never know if the things that they're saying are actual reality” or a plausible fabrication. This uncertainty imposes a “shadow workload” of hypervigilance that is often more cognitively taxing than manual documentation.

Moreover, this scepticism is reinforced by a deep institutional memory of failed automation. Veteran clinicians contextualised the current wave of AI tools within historical cycles of administrative overreach, arguing that “those of us who were around in the 90 s know HMOs tried to replace physicians with a rudimentary AI and failed.” This historical cynicism acts as a powerful barrier to durability, framing the AI not as a neutral assistant, but as the latest iteration of a failed managerial project.

The crisis of legitimacy also extends outward to the patient interaction. Durability is compromised when the technology introduces social friction into the consultation, with users observing that “research suggests patients are actually more skeptical of medical advice given by AI.” Consequently, the clinician is forced to perform “repair work” not only on the text but also on the patient relationship itself, further eroding the willingness to sustain use over time.

## 4 Discussion: The Paradox of Durability

This study challenges the prevailing narrative that the technical efficiency of AI documentation automatically translates into sustainable clinical value. While a substantial body of recent literature demonstrates that AI scribes can reduce documentation time and improve immediate workflow perceptions [10, 11, 12, 13]. Our netnographic analysis reveals that these proximal gains often fail to survive the transition to routine, long‐term practice. We identify three socio‐organisational mechanisms, specifically Incentive Conversion, Maintenance Entropy, and Legitimacy Collapse, which explain why “successful” pilots frequently degrade into “failed” durability.

### 4.1 Beyond the Jevons Paradox: From Relief to Throughput

Our identification of Incentive Conversion, defined as the process where time savings are captured by administrative throughput targets rather than clinician relief, offers a critical corrective to the existing evidence base. The current literature is heavily weighted towards short‐term pre‐post evaluations which consistently report reductions in burnout and cognitive load [14, 15]. Olson et al. [15] documented improvements in exhaustion and after‐hours work in a 30‐day multicentre evaluation. Our findings suggest that these studies capture a transient “honeymoon period” before institutional incentives realign [15].

We argue that the “efficiency” measured in these studies is liquid rather than stable. Kanaparthy et al. [16] hint in their rapid synthesis that productivity metrics often remain unchanged even as documentation time falls, suggesting a misalignment between clinician wellbeing and organisational goals [16]. Our data extends this observation by populating the mechanism: the demonstration of capacity to see more patients allows the “time saved” to be re‐allocated to volume through the Jevons Paradox. Consequently, the long‐term trajectory of AI adoption may not be a reduction in burnout, as suggested by Albrecht et al. (2025), but an intensification of labour density that existing short‐term study designs are structurally unable to detect [17].

### 4.2 The Invisible Labour of Maintenance Entropy

Our concept of Maintenance Entropy challenges the reliance on “time‐in‐note” metrics as a proxy for success. Quantitative studies report impressive reductions in EHR interaction time [18, 19], but they frequently miss the “shadow work” of integration that re‐emerges over time. Our findings show that the seamlessness of the pilot gives way to friction, including broken templates, context errors, and the need for manual re‐assembly, as vendor support withdraws.

This aligns with emerging critical commentaries which caution that automation does not eliminate labour but merely redistributes it [20, 21]. Van Tiem et al. [22] noted that generated documentation rarely adheres neatly to institutional norms, necessitating corrective work [22]. We interpret this not as a “usability” issue to be fixed by better prompts, but as a structural fiscal liability. This unpriced articulation work, as conceptualised in infrastructure studies, can accumulate as ongoing maintenance and integration demands, and may erode net efficiency benefits beyond early evaluation windows, even where retrospective cohorts report reductions in EHR and note time [19, 23]. Durability fails because the “cost” of the tool includes this invisible labour, which is absent from the initial Return on Investment (ROI) calculation.

### 4.3 Legitimacy Collapse and the Limits of Oversight

Finally, our analysis helps explain the fragility of clinician trust reported in the literature. Surveys often show high satisfaction [17], yet they also reveal a persistent undercurrent of scepticism regarding clinical fidelity [18]. We conceptualise this dynamic not merely as “mixed feedback” but as a mechanism of Legitimacy Collapse.

Current safety literature emphasises the need for “human‐in‐the‐loop” verification to manage hallucinations [24]. Our findings suggest that clinicians possess a finite verification budget. When the cognitive load required to monitor and correct AI outputs exceeds this limit, the tool loses institutional legitimacy. This helps explain findings from a recent evaluation in which high levels of clinician satisfaction coexisted with intensive oversight [18]. Over time, this oversight evolves into a sustained second shift of liability management, a dynamic described as the moral crumple zone [25]. As a result, the current literature is likely to overestimate the long‐term viability of these tools in high‐risk clinical environments because it does not adequately account for the cumulative fatigue associated with ongoing verification.

### 4.4 Ethical Implications of Verification and Epistemic Responsibility

Our finding of Legitimacy Collapse also raises an ethical question concerning the sustainability of human oversight. Participants described AI‐generated documentation as requiring persistent checking because plausible outputs could not always be assumed to represent the clinical encounter accurately. This produces what we conceptualise as a finite verification budget, where reviewing and correcting generated text becomes substantive clinical work rather than a negligible final check. Emerging empirical evidence supports this interpretation. Clinicians edit AI‐generated notes not simply for style, but to correct symptoms, diagnoses, orders, missing information and expressions of diagnostic certainty, while also responding to medicolegal and liability concerns, placing verification within clinical reasoning and professional risk management rather than ordinary proofreading [26, 27]. Controlled evaluations similarly show that generated notes can contain frequent omissions and clinically consequential errors, while omission errors may be particularly difficult to identify because their detection requires clinicians to recall information that is absent from the generated record [28, 29]. Our findings therefore suggest that the ethical adequacy of human oversight cannot be inferred merely from the requirement that a clinician reviews and signs the final note.

Our findings also add an operational dimension to arguments characterising AI scribes as epistemic agents. These systems do not merely reproduce spoken language, but select, organise and reformulate information that subsequently becomes part of the clinical record, thereby participating in the production of clinically consequential knowledge [30]. Professional responsibility for determining whether the resulting record is accurate and complete nevertheless remains substantially with the clinician [31]. Our earlier analysis of patient‐facing AI‐generated documentation identified a related accountability tension, where mandatory clinician sign‐off coexisted with productivity pressures, plausible errors and limited provenance mechanisms, making formal responsibility difficult to translate into meaningful control [32]. The present findings extend that argument by locating the problem within a durability mechanism. When confidence in generated output declines, clinicians do not relinquish responsibility for the record. Instead, accountability is exercised through increasingly intensive checking, correction, and reconstruction. Legitimacy Collapse therefore exposes an asymmetry between increasingly distributed production of the clinical record and continuing concentration of responsibility for validating that record.

Our findings further complicate the assumption that retaining a human in the loop is, by itself, an adequate safeguard. Ethical analyses of generative clinical documentation emphasise clinician review as protection against bias and error propagation and their potential effects on clinical judgement [33]. Yet meaningful scrutiny requires more than formal sign‐off. It depends on clinicians having sufficient time, attention and access to encounter evidence to determine whether generated statements are accurate and whether clinically important information has been omitted. This is particularly important because omission creates a distinctive verification problem. Clinicians may need to reconstruct the encounter mentally to recognise information that is absent, rather than simply identify an incorrect statement that is present, and this task may become more difficult when review occurs after the encounter [29]. Similar concerns arise where clinically relevant errors remain embedded within otherwise usable documentation, while existing evidence does not establish that routine clinician review can reliably identify and remedy such errors [28, 31]. Our verification‐budget finding adds a temporal dimension to this problem. Human oversight may remain procedurally present while the practical capacity required to exercise that oversight becomes increasingly strained as verification demands accumulate.

The ethical significance of Legitimacy Collapse therefore lies in the distinction between formal accountability and supported accountability. Requiring clinicians to approve AI‐generated documentation preserves a visible locus of responsibility, but it does not ensure that the conditions required to exercise that responsibility remain available. Recent ethical work similarly argues that clinician review should not be treated as a sufficient standalone protection, particularly where proofreading burden, productivity expectations and organisational arrangements may weaken effective scrutiny, and it calls for protected time and clearer allocation of responsibility across clinicians, institutions and vendors [31]. Our findings extend this argument by identifying how deteriorating epistemic trust may progressively increase the work required to discharge retained professional responsibility. As confidence declines, verification becomes more intensive, while the additional effort required to sustain safe use may further erode the value obtained from the technology. Legitimacy Collapse may therefore occur before technical failure or formal abandonment, when the cognitive demands of maintaining confidence in generated documentation begin to strain the clinician's available verification capacity.

This interpretation does not imply that human verification should be reduced or removed. Rather, it suggests that meaningful oversight is an organisational achievement rather than a fixed property of system design. The present study does not establish a measurable point at which verification becomes ineffective, nor can it determine whether verification fatigue results in unsafe documentation. It does, however, identify a mechanism through which a formally compliant human‐in‐the‐loop arrangement may become ethically fragile over time. The ethical problem is therefore not that clinicians retain responsibility for AI‐generated documentation, but that responsibility may remain concentrated with clinicians while the epistemic and organisational capacity required to discharge that responsibility progressively deteriorates. The durability of AI‐enabled documentation depends partly on whether continuing responsibility for the clinical record is accompanied by durable capacity to scrutinise, correct and, where necessary, reject the outputs through which that record is produced.

### 4.5 Governance Implications for Fiscal Credibility and Ethical Accountability

The distinction between formal and supported accountability raises a practical question about where judgements on the continuing use of AI‐enabled clinical documentation should be made. We recommend that fiscal oversight and ethical oversight be constituted as separate and independent functions, rather than centralised within a single governance structure. Fiscal oversight would assess procurement and renewal costs, realised efficiency dividends, maintenance expenditure and the durability of net institutional value. Ethical oversight would assess clinical safety, the feasibility of meaningful verification, the allocation of professional responsibility and the effects of altered workloads on patient care. Each function would hold its own terms of reference, membership, evidence review and reporting line. The two functions should maintain independent evaluative authority while sharing relevant evidence and coordinating periodic reviews. Their respective assessments should be presented without alteration to the executive or board committee accountable for deployment decisions (Figure 1). Separation in this sense concerns judgement rather than administration. It does not require new organisations.

**Figure 1:**
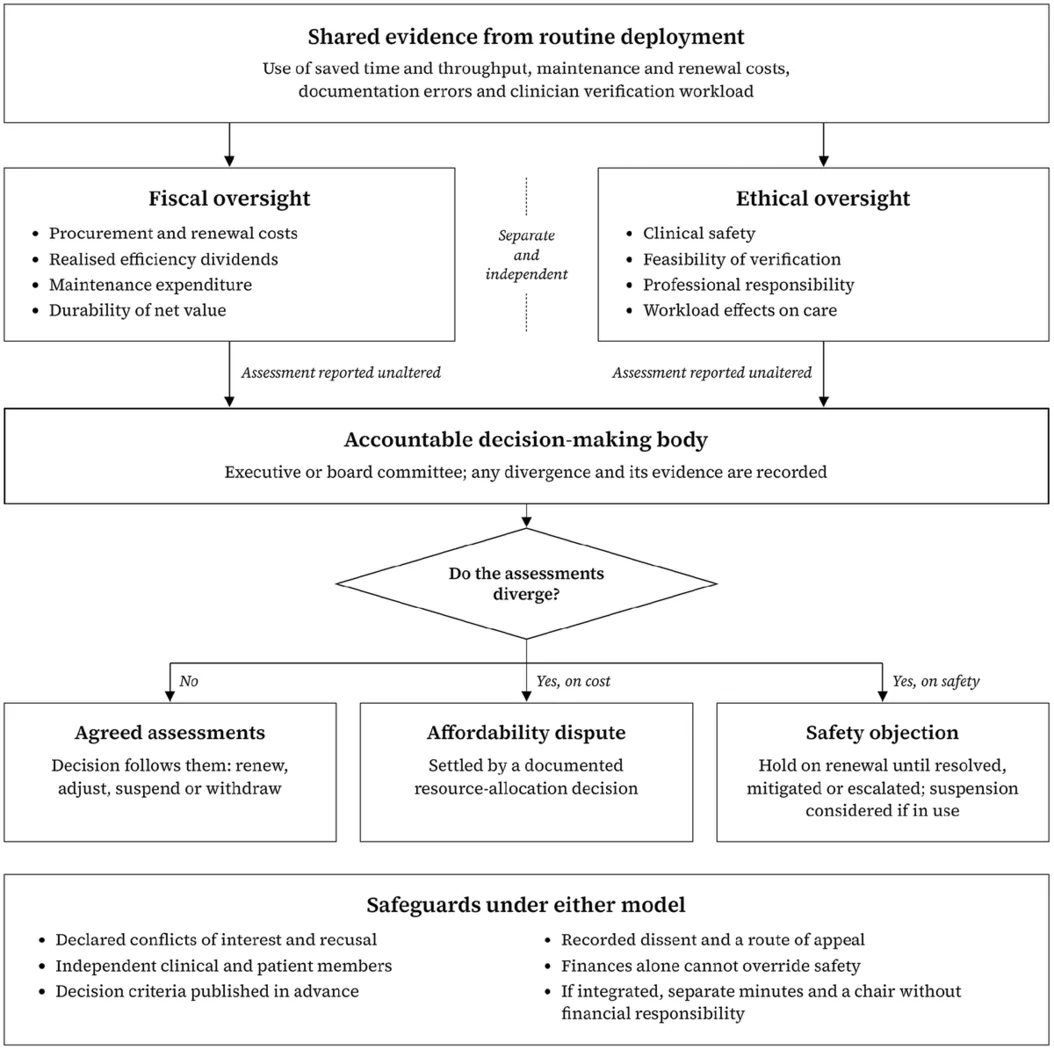
Proposed separation of fiscal and ethical oversight for AI‐enabled clinical documentation. Each function reports its assessment unaltered to the accountable decision‐making body. Divergence on affordability is settled through a documented resource‐allocation decision, whereas an unresolved safety objection places a conditional hold on renewal or expansion and prompts consideration of suspension where the tool is already in use. Safeguards apply under either a separate or an integrated model.

The proposal extends, rather than departs from, existing approaches to governing clinical AI. Reddy et al. [34] describe a governance model in which a clinical governance committee, drawing on clinicians, managers, patient representatives and ethics expertise, oversees the introduction and deployment of AI and reports to an accountable clinical owner. International guidance similarly treats clear lines of responsibility and accountability as a condition of ethical AI use in health [35]. In the NHS, ethical oversight could build on the clinical risk management process that deploying organisations already maintain under a named Clinical Safety Officer [36], while fiscal oversight would remain within finance and procurement governance.

An analysis of 74 official guidance documents on AI scribes found that organisational safety cases, vendor due diligence and data protection assessments narrow product‐level uncertainty, yet leave undefined the encounter‐level support clinicians need to detect hallucination and omission [37]. What our analysis adds is an argument that these routes should stay independent after a deployment becomes routine. Most governance attention falls on approval. The mechanisms we identified emerge later, once the conditions under which approval was granted have changed, and it is at this stage that the independence of ethical judgement is most likely to be tested.

Our rationale follows from the mechanisms identified in the analysis. Incentive Conversion suggests that apparent financial gains may reflect the transfer of time savings into increased throughput rather than sustained workforce relief. Maintenance Entropy points to costs that are easily omitted from initial financial calculations, and Legitimacy Collapse indicates that formal clinician sign‐off may persist after the practical capacity for scrutiny has weakened. In each case, evidence that a deployment is succeeding financially may conceal ethical deterioration. An organisation that has procured a technology also holds a stake in its apparent success, and work on institutional conflicts of interest recommends that decisions affected by such a stake be overseen by people who do not share it [38]. A single structure would ask one body to judge both. Integration may appear more efficient. In our view it would reproduce, at the level of governance, the asymmetry we observed at the level of the clinician, in which responsibility for safety is formally retained while the conditions for exercising it are subordinated to productivity [25, 31].

Conflicts between the two assessments should be expected rather than treated as failures of process. Divergence is itself informative, since it may signal that projected savings rest on clinical work, such as verification, that fiscal indicators do not capture. Where evaluations diverge, the disagreement, its evidential basis and the proposed response should be recorded before any decision on procurement renewal, expansion or major workflow change. Projected savings accompanied by an unsustainable verification workload, for example, should prompt further clinical assessment and consideration of workload adjustment, revised deployment conditions or suspension of expansion. Disputes about affordability alone may be settled through documented resource‐allocation decisions.

Unresolved material safety concerns are different. We propose that an ethical or clinical safety objection should operate as a conditional hold, so that the deployment cannot be renewed or extended until the objection has been resolved, accepted with mitigation by the accountable clinical safety lead, or referred through existing organisational escalation procedures. Where the concern affects a deployment already in use, the same clinical safety lead should determine whether immediate mitigation, temporary suspension or further escalation is required under established institutional procedures. Financial performance should not be admissible as sufficient grounds for overriding such an objection. These procedures follow the logic of accountability for reasonableness, under which the grounds for limit‐setting decisions should be public, relevant to those affected and open to challenge and revision [39].

Objective decision‐making depends on safeguards under either model. Where the functions are separate, as we recommend, these include independent clinical and patient representation within ethical oversight, declared and managed conflicts of interest with recusal where a member holds a relevant financial interest [38], decision criteria published before review, shared access to evidence on documentation errors and verification workload, and a recorded route for appeal. Some institutions may still retain a single integrated committee for reasons of scale or existing structure. Additional protections would then be needed. Fiscal and clinical safety findings should be reported and minuted separately, the chair should not hold direct responsibility for the financial performance of the deployment, and dissenting safety assessments should be recorded rather than absorbed into a consensus judgement. Under both models, the purpose is to prevent the expected savings of a technology from shaping the assessment of its clinical risks. The proposed separation is a normative recommendation derived from the durability mechanisms identified here, not an empirically validated institutional model, and precise decision authority will vary with institutional and regulatory requirements.

## 5 Strengths and Limitations

This study makes a distinctive contribution to the digital health literature, shifting the analytical lens from short‐term efficiency metrics to the longitudinal mechanisms of durability. Netnography enabled us to capture the backstage discourse of clinical work, revealing candid and unmonitored frustrations that are often sanitised in formal surveys or vendor‐sponsored case studies. This methodological approach supports the identification of emergent phenomena, such as Incentive Conversion, which are structurally invisible to standard before‐and‐after quantitative evaluations. The analysis also offers a theoretical vocabulary for explaining why technically functional tools fail to survive in real‐world settings, moving beyond simplistic attributions of user resistance.

Several limitations should be acknowledged. First, as with all netnographic research, the data represent a purposive sample of individuals sufficiently motivated to discuss their professional experiences in online communities. This may introduce a negativity bias, since users are historically more likely to document friction and failure than seamless function. Second, although rigorous verification protocols were employed to exclude non‐clinical voices, the platform's anonymity precludes independent validation of employment status or specific health system identity. Finally, the analysis is qualitative in nature. Although we identify specific mechanisms of value decay, we cannot quantify the prevalence of these phenomena across the wider health system without further mixed‐methods research. Future scholarship would benefit from longitudinal cohort studies that track productivity targets and maintenance costs over multi‐year periods, validating these findings at scale. The study did not compare governance arrangements or establish the effectiveness of independent fiscal and ethical oversight; the proposed institutional safeguards should therefore be evaluated in future implementation research.

## 6 Conclusion

The central finding of this study is that digital health failure is fundamentally a problem of value translation rather than technical innovation. We demonstrate that even when value is demonstrable in the pilot phase and governable in the procurement phase, it frequently fails to become durable in the routine phase. Our findings reveal that the sustainability of AI documentation is undermined not by algorithmic error, but by an extractive institutional logic. Through the mechanism of Incentive Conversion, health systems appear to systematically cannibalise the efficiency gains of the technology, transforming potential workforce relief into increased throughput pressure. Simultaneously, the unpriced labour of Maintenance Entropy and the cognitive burden of Legitimacy Collapse create a maintenance debt that eventually exhausts the user's willingness to sustain the intervention.

The implications for policy and health system leadership are significant. For AI documentation technologies to become fiscally credible public programmes, policymakers must move beyond a pilot‐to‐purchase mindset and adopt a lifecycle management approach. First, to prevent the erosion of clinician trust, efficiency dividends must be contractually ring‐fenced. Time saved by AI should be formally allocated to non‐clinical recovery or care quality, rather than being automatically reabsorbed into slot utilisation. Second, procurement budgets must explicitly account for the ongoing articulation work required to bridge the gap between AI outputs and the electronic health record, acknowledging that integration is a continuous labour rather than a one‐time capital expense. Finally, the verification budget of clinicians must be respected within productivity models. Liability frameworks and workload calculations should acknowledge that human‐in‐the‐loop oversight is a form of labour, and this cognitive load must be factored into realistic performance targets. Responsibility for these recommendations should be divided between distinct oversight functions. Ring‐fencing efficiency dividends and pricing maintenance labour fall to fiscal oversight, whereas protecting the verification budget and judging whether clinician review remains meaningful fall to ethical oversight. We recommend that the two functions remain separate and independent, each reporting its assessment unaltered to the accountable decision‐making body, with documented escalation where they conflict and no clinical safety objection overridden on financial grounds alone.

Ultimately, durability is not an intrinsic property of the software but a property of the socio‐technical contract between the institution and its workforce. Unless health systems shift from treating AI as a mechanism for extraction to treating it as a mechanism for sustainability, these tools will remain fiscally fragile, powerful in the pilot phase but powerless to survive the complex reality of routine clinical practice.

## Funding

The authors have nothing to report.

## Disclosure

The authors have nothing to report.

## Ethics Statement

Ethical approval for this study was obtained from Aston University.

## Consent

The study analysed publicly available online data and did not involve direct interaction with participants.

## Conflicts of Interest

The authors declare no conflicts of interest.

## Data Availability

The data analysed in this study are derived from publicly accessible online discussions. In line with ethical approval and to minimise risks of re‐identification, the curated datasets and harvested text segments are not publicly shared. Further details about the data collection and analytic procedures are available from the corresponding author upon reasonable request.
